# Creatine and Resistance Training: A Combined Approach to Attenuate Doxorubicin-Induced Cardiotoxicity

**DOI:** 10.3390/nu15184048

**Published:** 2023-09-19

**Authors:** David Law, Mitchel A. Magrini, Jacob A. Siedlik, Joan Eckerson, Kristen M. Drescher, Eric C. Bredahl

**Affiliations:** 1 Department of Exercise Science and Pre-Health Professions, Creighton University, 2500 California Plaza, Omaha, NE 68178, USA; davidlaw@creighton.edu (D.L.); mitchelmagrini@creighton.edu (M.A.M.); jakesiedlik@creighton.edu (J.A.S.); joaneckerson@creighton.edu (J.E.); 2 Department of Medical Microbiology and Immunology, Creighton University, Omaha NE 68178, USA; kristendrescher@creighton.edu

**Keywords:** creatine, exercise, cancer, cardiac dysfunction

## Abstract

Doxorubicin (DOX), a potent chemotherapy agent, useful in the treatment of solid tumors, lymphomas, and leukemias, is limited by its potentially lethal cardiotoxicity. However, exercise has been consistently shown to mitigate the side effects of DOX, including cardiotoxicity. To date, most studies examining the relationship between exercise and DOX-induced cardiotoxicity have focused on aerobic exercise, with very few examining the role of anerobic activity. Therefore, this investigation explored the potential of creatine (CR) and resistance training (RT) in preserving cardiac health during DOX therapy. Male Sprague-Dawley rats were grouped into RT, RT + CR, sedentary (SED), and SED + CR, with each division further branching into saline (SAL) or DOX-treated subsets post-10 weeks of RT or SED activity. RT comprised progressive training utilizing specialized cages for bipedal stance feeding. CR-treated groups ingested water mixed with 1% CR monohydrate and 5% dextrose, while control animals received 5% dextrose. At week 10, DOX was administered (2 mg/kg/week) over 4-weeks to an 8 mg/kg cumulative dose. Cardiac function post-DOX treatment was assessed via transthoracic echocardiography. Left ventricular diameter during diastole was lower in DOX + CR, RT + DOX, and RT + CR + DOX compared to SED + DOX (*p* < 0.05). Additionally, cardiac mass was significantly greater in RT + CR + DOX SED + DOX animals (*p* < 0.05). These results suggest RT and CR supplementation, separately and in combination, could attenuate some measures of DOX-induced cardiotoxicity and may offer a cost-effective way to complement cancer treatments and enhance patient outcomes. More investigations are essential to better understand CR’s prolonged effects during DOX therapy and its clinical implications.

## 1. Introduction

Doxorubicin (DOX, trade name Adriamycin) is a potent anthracycline chemotherapy agent used to treat a variety of cancers such as leukemias, lymphomas, breast cancer, soft tissue sarcomas, and lung cancer [1]. Although DOX is an effective treatment for cancer, it does come with significant side effects like hyperpigmentation, fatigue, alopecia, neutropenia, edema, and kidney dysfunction [1]. DOX is also associated with dose-dependent cardiotoxicities [1,2,3,4], with a maximal clinical dose of 550 mg/m^2^ [5]. Therefore, despite its usefulness in the treatment of cancer, DOX may increase the risk of potentially life-threatening cardiomyopathies which, in turn, adversely affect quality of life (QOL) and the ability to perform activities of daily living (ADL) [6,7]. 

At the cellular level, free radicals generated by DOX treatment, such as superoxide, decrease the activity of mitochondrial creatine kinase (CK) and the eventual loss of the myocardium, which results in a decline in cardiac function [8]. Furthermore, the reactive oxygen species (ROS) generation caused by DOX can impact the interplay between CK and Ca^2+^-ATPase required for the resequestration of calcium into the sarcoplasmic reticulum and, ultimately, harm the cardiac contraction cycle. Under normal conditions, this coupling is mediated by the phosphorylated CR produced by CK helping to turnover ADP to ATP to facilitate Ca^2+^ reuptake [9]. However, a continuously high Ca^2+^ level in the absence of reuptake leads to the activation of a calcineurin signaling pathway, resulting in cardiac pathologies [10] which include dilated cardiomyopathy and heart failure [11,12].

Previous studies have shown that aerobic exercise can minimize DOX-induced cardiotoxicity [12,13]. For example, it was demonstrated in perfused rat models that an 8-week long course of voluntary running caused a reduction in the cardiotoxic impact of DOX [14]. Other studies have also reported that both acute and long-term aerobic exercise can attenuate the side effects of DOX treatment, particularly those related to DOX cardiotoxicity [13,15,16,17]. Furthermore, aerobic exercise has been shown to increase the expression of antioxidants [18], the abundance of heat shock proteins [19], and the upregulated mitochondria-specific ATP-binding cassette [20], and can ameliorate the increase in autophagosomes [21]. On the clinical side, it has been shown that exercise has a positive influence on metrics of physical and mental health in breast cancer and non-breast cancer patients [22,23], as well as cancer survivors, and improves QOL [24]. Although exercise most certainly plays a protective role in minimizing the side effects of DOX treatment, the majority of studies have focused on aerobic exercise [13,15,18,25], with very few examining the effect of anerobic or resistance training (RT) as an adjuvant therapy in a basic science setting. This study seeks to build upon previous works to further illuminate the role of RT in mitigating the effects of DOX-induced cardiotoxicity. While the processes of exercise-induced hypertrophy versus development of pathological cardiomyopathies are distinct, they do interact with common elements of the IGF_1_-PI_3_K-Akt signaling pathway [11,12,26], leading to the inhibition of cell death signaling, the promotion of cardiomyocyte survival, and the activation of genes that result in increased cell size. This process, termed physiological hypertrophy [26,27], ultimately prevents the death of cardiomyocytes and preserves cardiac tissue. It is also hypothesized that this pathway can inhibit mechanisms related to cardiomyocyte death more directly, but that requires further study [11,12]. Therefore, it seems plausible that the preservation of cardiac muscle mass is possible via RT and may attenuate the degree of DOX-induced cardiotoxicity. 

Although exercise has been shown to minimize several of the cytotoxic effects of DOX treatment, it may not be a viable option for all patients. In this case, another potential adjuvant therapy could be the dietary supplement CR, which has previously been shown to have a therapeutic role in several disease states characterized by muscle atrophy, neurological decline, and metabolic dysfunction [28]. In addition to these clinical applications, CR has been shown to have an impact in reducing muscle fatigue caused by DOX administration [29] and may be able to be used against the more cardiotoxic side effects of DOX. Specifically, CR has been shown to minimize DOX-induced cytotoxicity in cultured myocytes [30]. CR supplementation has also been shown to have antioxidant effects [31,32], which could attenuate the excessive generation of ROS caused by DOX, and may help impede the decline in cardiac function by preventing the free radical-mediated deactivation of CK. Additionally, supplemental CR serves as a substrate for CK, potentially recoupling it to Ca^2+^-ATPase, and thus preventing the activation of the calcineurin pathway and the development of subsequent cardiomyopathies.

The objective of this investigation was to better understand the capacity of CR and RT, both separately and in combination, to preserve cardiac muscle function during DOX treatment. We hypothesized that a combined treatment of CR and RT would preserve cardiac muscle function to a greater degree than either treatment alone. We have recently demonstrated that CR has the ability to minimize rates of necrosis and apoptosis in DOX-treated myoblasts in vitro [30]. If effective, the use of CR and RT could have a positive clinical impact because they represent a simple and low-cost adjunctive therapy that could benefit patients undergoing all stages of DOX treatment.

## 2. Methods

### 2.1. Animals

All procedures and data collection were performed in accordance with a Creighton University IACUC-approved protocol. Ten-week-old male Sprague-Dawley rats (N = 80) were kept on a 12:12 h light and dark cycle and were housed two per cage with food (Envigo #2018 Global 18% Rodent Diet, Envigo, Indianapolis, IN, USA) and water provided ad libitum. At the start of the study, the animals were assigned to one of the following groups: RT (N = 20), RT + CR (N = 20), sedentary (SED, N = 20), or SED + CR (N = 20) group. Following a 10-week training period, the animals in each group were subdivided into a saline (SAL) or DOX-treated group. RT + SAL (N = 10), RT + DOX (N = 10), RT + CR + SAL (N = 10), RT + CR + DOX (N = 10), SED + SAL (N = 10), SED + DOX (N = 10), SED + CR + SAL (N = 10), or SED + CR + DOX (N = 10). 

### 2.2. Creatine and Doxorubicin Administration

Animals assigned to the CR-treated groups received a 1% CR monohydrate (Sigma Aldrich, St. Louis, MO, USA)/5% dextrose (Sigma Aldrich, St. Louis, MO, USA) solution administered into the drinking water and consumed ad libitum. This dose has previously been shown to significantly increase the total intracellular CR and phosphocreatine (PCR) content within rat fast twitch tibialis anterior muscle fibers [33] and corresponds to an average of 1.6 g/day for a 360 g rat consuming 50–70 mL of fluid per day [33]. Although daily administration of an exact CR/dextrose dose would have been possible via gavage, it is a high-stress procedure and not feasible for long-term studies. Thus, we elected to provide CR in a continual manner throughout the study. Animals who were assigned to a DOX treatment group received weekly intraperitoneal injections of DOX (2 mg/kg) with a cumulative dose of 8 mg/kg to replicate a clinical dosing regimen. The DOX treatment was prepared from a 2 mg/mL stock solution (Vibrac, Westlake, TX, USA). Animals in SAL groups were treated with an equivalent (calculated in the same manner as DOX) dose of 0.9% saline (Thermo Scientific, Waltham, MA, USA). At high concentrations, DOX-induced cardiotoxicity becomes apparent in a few days, with an extremely low survival rate; however, if DOX is given in a serial manner (i.e., weekly injections), the survival rate improves significantly while still showing cardiotoxicity [34]. Furthermore, the DOX dose and treatment schedule for this study was selected to recreate a clinically relevant treatment condition. 

### 2.3. Training Protocol

To provide a representation of low intensity RT, animals assigned to the RT groups were placed in specialized cages, requiring the animals to rise onto their hind limbs to access their food and water, as described by Yao et al. [35]. Forcing rats to attain a bipedal stance for feeding and drinking increases hind limb skeletal muscle mass when compared to normal cage activity [35,36]. Furthermore, this model is less stressful than the alternative methods for stimulating resistance (i.e., electrical stimulation and weighted ladder climbing). The RT model was employed in a progressive manner by starting the training sessions at a height of 28 cm for 3-days and thereafter, the cage height was raised 2.5 cm every third day until the cage height reached 35.5 cm which was maintained for the remainder of the 10-week training period.

### 2.4. Cardiac Analysis

To gain a better understanding of the combination of CR and RT on DOX-induced cardiac muscle dysfunction, this study utilized transthoracic echocardiography as an in vivo method of assessing cardiac function. Prior to echocardiography, the animals were placed under anesthesia with inhaled 95% oxygen/5% isoflurane until the end of the procedure. Proper sedation was verified by a toe and tail pinch. Once the animal was properly sedated, the abdomen was shaved using a commercially available pair of hair clippers. Cardiac images were obtained using an ultrasound machine (General Electric Healthcare, Madison, WI, USA) to measure the following variables: left ventricular dimension during systole (LVDS), or the distance between septal and posterior walls at end systole; left ventricular dimension diastole (LVDD), or the distance between septal and posterior walls at end diastole; septal wall thickness at systole (SWTS), or the ventricular wall at top of image at end systole; septal wall thickness at diastole (SWTD), or the ventricular wall at top of image at end diastole; posterior wall thickness at systole (PWTS), or the ventricular wall at bottom of image at end systole; posterior wall thickness at diastole (PWTD), or the ventricular wall at bottom of image at end diastole. Each variable was measured in triplicate and the average was used for statistical analyses.

### 2.5. Statistical Analysis

All data are presented as mean ± standard deviation (SD). Prior to DOX treatment, the data were analyzed using a two-way (exercise × supplement) analysis of variance (ANOVA). Post-DOX treatment, the data were analyzed using a three-way ANOVA (exercise × supplement × drug). If a significant main effect or interaction was observed, Tukey’s post hoc analysis was used to determine significant differences between conditions. Graph Pad Prism (San Diego, CA, USA) was used for all statistical analyses with a significance level of α = 0.05.

## 3. Results

**Figure 1:**
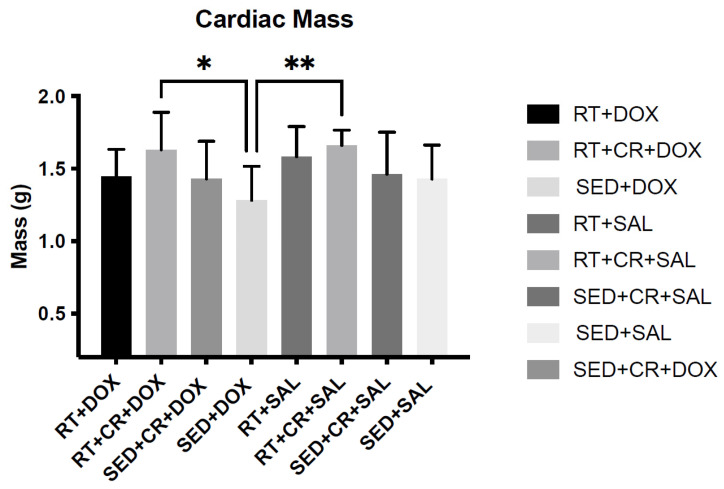
Cardiac mass post-doxorubicin (DOX) treatment and cardiac mass post-DOX treatment. Asterisks indicate a significant difference from SED + DOX, * (*p* < 0.05), ** (*p* < 0.01).

Body mass (BM) was not significantly different among the groups pre-training; however, after 10-weeks of RT, a significant main effect was observed for exercise. When compared to SED animals, RT animals were significantly heavier (*p* < 0.05). Additionally, RT + CR animals were also significantly heavier than those in the SED condition (*p* < 0.05). Pre- and post-training BM can be seen in Table 1. Following DOX treatment, a significant (*p* < 0.05) main affect for drug was observed for BM and post hoc testing revealed no significant differences across the groups. It should be noted that DOX-treated animals exhibited varying degrees of ascites, which would impact their BM. When cardiac mass was analyzed, there were significant main effects for both exercise and supplement (*p* < 0.05). Post hoc testing revealed a significant difference in cardiac mass between SED + DOX (1.28 ± 0.23 g) and RT + CR + DOX (1.63 ± 0.25 g), and between SED + DOX and RT + SAL + CR (1.66 ± 0.25 g) (Table 2). Differences in cardiac mass can also be seen in Figure 1.

### Cardiac Function

**Figure 2:**
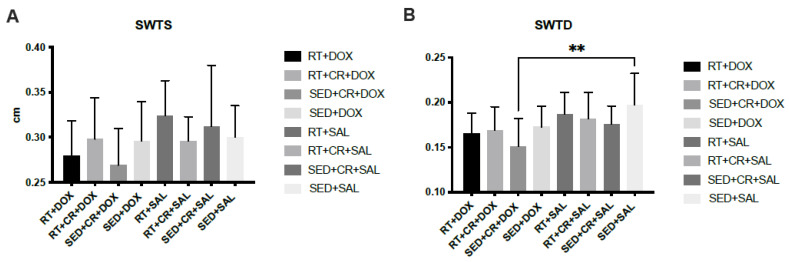

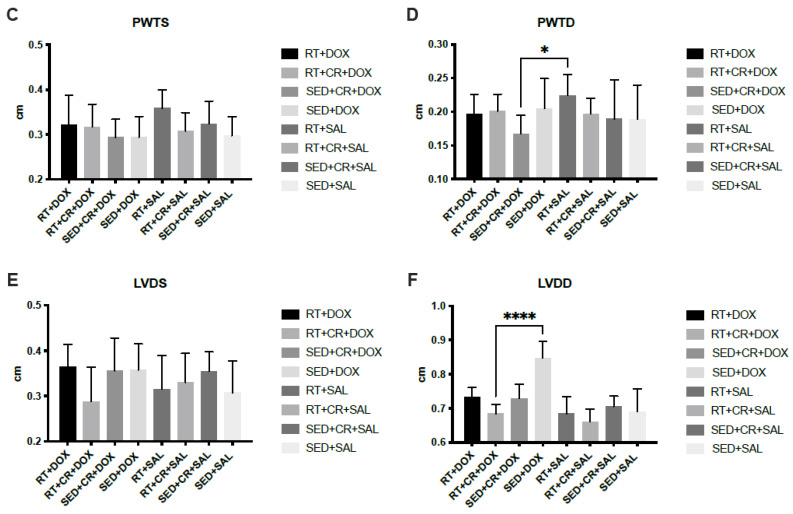
Septal wall thickness during systole (SWTS, (**A**)), septal wall thickness during diastole (SWTD, (**B**)), posterior wall thickness during systole (PWTS, (**C**)), posterior wall thickness during diastole (PWTD, (**D**)), left ventricular diameter during systole (LVDS, (**E**)), and left ventricular diameter during diastole (LVDD, (**F**)). Symbols indicate a significant difference,* (*p* < 0.05), ** (*p* < 0.01), **** (*p* < 0.0001).

The results for cardiac analysis (Figure 2) showed that SED + CR + DOX, RT + DOX, and RT + CR + DOX all demonstrated significantly lower LVDD compared to SED + DOX (*p* < 0.05). There was also a significant difference in LVDD between SED + CR + DOX and RT + CR + SAL (*p* < 0.05). The results for PWTD showed that there was a significant difference between SED + CR + DOX and RT + SAL (*p* < 0.05). SWTD showed a significant difference between SED + CR + DOX and SED + SAL (*p* < 0.05). There were no significant interactions or main effects between groups for PWTS, SWTS, and LVDS. 

## 4. Discussion

DOX is one of the most widely used chemotherapeutic agents and is a highly effective treatment for a variety of cancers [17,37,38]; however, it causes a number of severe side effects that can greatly alter the patient’s QOL and ability to perform ADLs [39,40]. Although several investigations have shown that physical activity attenuates the adverse cardiovascular effects of DOX [41,42,43], the current study sought to build upon these investigations by using a more clinically similar DOX dosing schedule. In addition, this study used a low-intensity RT model designed to represent an active individual who continues their training program during chemotherapy.

When using DOX as a chemotherapy agent, a common limiting factor is the development of potentially lethal heart failure, which typically occurs in a dose-dependent manner [44]. The increased chamber diameter and loss of cardiac dimensions reduce ejection fraction, typically resulting in the cessation of DOX administration and leaving the patient with a potentially serious heart condition [45,46]. The ability to preserve chamber diameter during DOX treatment could increase the duration and dose of DOX that can be used in clinical settings and/or improve patient outcomes. Therefore, the preservation of LVDD found in this study following 10-weeks of RT and CR has clinical significance since it may help to attenuate DOX-induced heart failure. Furthermore, given that there were no significant differences between RT + DOX, SED + CR + DOX, and RT + CR + DOX for LVDD, it appears that CR supplementation or RT alone may attenuate DOX-induced changes in LVDD. Although the preservation of LVDD is meaningful, this study did not observe any further changes in cardiac function. The goal of this study was to replicate the conditions experienced by a patient undergoing DOX treatment. As such the DOX dose used in this study was relatively small and given over a series of weeks, which, we believe, was not sufficient to induce the same level of DOX-induced cardiotoxicity as other investigations. Despite this lower level of dysfunction, there was significant preservation of cardiac mass with a combination of RT and CR during DOX treatment, which suggests that maintenance of the myocardium may be possible; however, further studies are warranted.

Previous studies from our laboratory have shown that ex vivo incubation of excised rat skeletal muscle with CR prior to DOX treatment preserved skeletal muscle function, that RT prior to DOX treatment can attenuate DOX-induced muscle dysfunction, and that CR can minimize cell death in cultured myocytes exposed to DOX [30,43,47,48,49,50]. This study sought to build upon these findings by assessing the interaction between CR and RT in the whole animal. Given the interplay between the atrophy and hypertrophy signaling cascades in the IGF_1_-PI_3_K-Akt pathway [11,12,26], it is possible that RT could slow cell death signaling and promote cardiomyocyte survival. Ultimately, we postulate that RT can offset the loss of the myocardium caused by DOX. However, it should be noted that this study employed RT prior to DOX treatment, which likely increased cardiac mass as a result.

Another core aspect of this study was the administration of CR during DOX treatment. Previous work has demonstrated that cardiomyocytes lose their ability to properly couple mitochondrially produced ATP to the recycling of PCR and ADP with a corresponding reduction in CR and CK following DOX exposure [51]. Additionally, DOX reduces CR transport, decreases V_max_, lowers K_m,_ and reduces CR transporter expression on the cell surface in cultured cardiomyocytes [52]. Given that CR inherently mitigates oxidative stress, it is also likely that this reduction in CR transport and processing also allows ROS to form more freely, thereby increasing the chances of cardiac damage and cardiomyopathy development. Thus, the increase in concentration of CR through supplementation could make up for this reduced transportation rate, mitigate oxidative stress damage in the heart, and help to preserve the strength of the myocardium as well as the health of other organs [29,53,54]. Since this transportation rate reduction may be long-term, this may also indicate a need for long-term CR supplementation after successful cancer elimination, although that will require further research. A major limitation of the oxidative-stress mitigating nature of CR is that it may limit oxidative stress at the site of cancer as well, thereby protecting the tumor from DOX. If this is the case, it may render CR impracticable for use during cancer treatment. To gain a better perspective, future studies need to focus on how CR may interfere with the cancer killing effects of DOX and cancer cell viability through a combination single cell and whole animal models. Additionally, more studies need to study how RT could impact the function of other tissues sensitive to DOX treatment.

## 5. Conclusions

The combination of RT and CR represent a low-cost and easily deployable adjunctive therapy to attenuate the decline in cardiac function during DOX treatment. However, this potential therapy requires more study to ensure that CR is not interfering with the cancer killing properties of DOX. The finding that RT alone provided cardioprotective effects also supports the incorporation of RT into treatment plans. Ultimately, this study helps to further highlight the role of exercise in minimizing chemotherapy-related side effects.

## Figures and Tables

**Table 1:** Body mass (mean ± SD) pre-training and post-training in grams (g). Asterisks indicate a significant difference from SED (*p* < 0.05).

	Pre-Training Body Mass (g)	Post-Training Body Mass (g)
SED	328.1 ± 11.6	427 ± 17.2
SED + CR	331.0 ± 16.04	433.8 ± 16.7
RT	323.6 ± 14.4	450.3 ± 28.9 *
RT + CR	322 ± 9.9	459 ± 20.4 *

**Table 2:** Body mass (mean ± SD) and cardiac mass post-doxorubicin (DOX) treatment and cardiac mass post-DOX treatment. Asterisks indicate a significant difference from SED + DOX (*p* < 0.05).

	Body Mass (g)	Cardiac Mass (g)
SED + SAL	431.1 ± 36.4	1.42 ± 0.23
SED + DOX	402.3 ± 14.6	1.28 ± 0.23
RT + SAL	444.6 ± 32.2	1.58 ± 0.2
RT + DOX	432.5 ± 56.2	1.44 ± 0.18
SED + CR + SAL	435.8 ± 32.3	1.46 ± 0.29
SED + CR + DOX	399.6 ± 51.1	1.42 ± 0.26
RT + CR + SAL	428.7 ± 22.8	1.66 ± 0.1 *
RT + CR + DOX	406.3 ± 38.9	1.63 ± 0.25 *

## Data Availability

Data available upon request.
